# Consistently doing what we know is right and important for “Medicine and Life”

**Published:** 2011-11-24

**Authors:** F Popa

**Affiliations:** Carol Davila University of Medicine and PharmacyRomania

In our consistently doing what we know is right and important for "Medicine and Life", we can express selfesteem and satisfaction after full honest effort with the successful carrying out of the First International Congress "Health – Food – Welfare" which took place at Aro Palace Hotel, Brasov, 15-17 October, 2011, under the patronage of the Romanian Patriarchate, The Romanian Academy, The Ministry of Public Health, The Ministry of Agriculture and Rural Development, The Ministry of Education, Research, Innovation and Sport in collaboration with the International Association of Distribution (A.I.D.A Bruxelles) and EHI Retail Institute. The quality of leadership has been demonstrated by the considerable willingness for cooperation proved by all the stakeholders. That is the reason why we are experiencing a real joy, because, as William Shakespeare said, joy’s soul lies in the doing.

**Photo 1:**
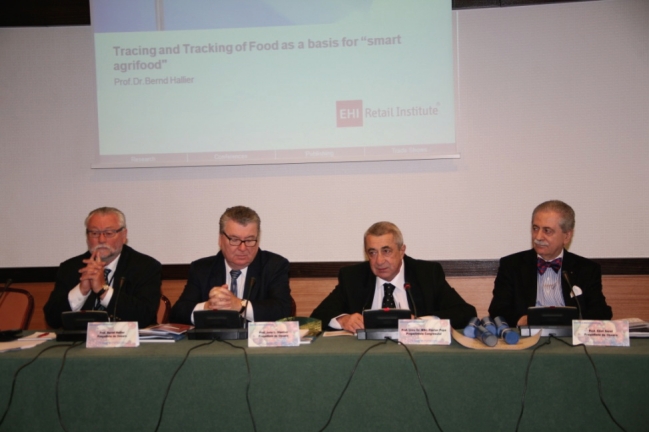
**The President of the Organizing Committee of the Congress, Professor Florian Popa,** with **Professor Eliot Sorel** (left-hand side), and **Professor Bernd Hallier, ** and **Professor John L. Stanton **on the right-hand side

As it is already well known, on that occasion a letter of intent had been signed (www.european-retailacademy.org/index.php, 17.10.2011: New Global Initiative) by **Professor Florian Popa, Rector of the "Carol Davila" University of Medicine and Pharmacy, Bucharest and President of the Organizing Committee, and the three Honorary Presidents, Professor Eliot Sorel/George Washington University/USA, Professor Bernd Hallier/Germany, Professor John L. Stanton/University of Philadelphia, USA** and other leading professors attending the event to cooperate in the future on the following topics: - *a Romanian Competence Center* for **Global good agricultural practices** (The GLOBALG.A.P standard is primarily designed to reassure consumers about how food is produced on the farm by minimizing detrimental environmental impacts of farming operations, reducing the use of chemical inputs and ensuring a responsible approach to worker health and safety as well as animal welfare) and **“smartagrifood”** *(Future Internet for Safe and Healthy Food from Farm to Fork; smart farming -* individual treatment of animals, plants or m2 of land at the right place and right time through sophisticated sensing & monitoring, decision support and precise application to improve efficiency, productivity, quality, flexibility and chain responsiveness; *smart agri-logistics* - is about intelligent matching demand and sourcing followed by smart transport and logistics of agri-food products by e.g. auto-identification, conditioned transport using sensors and control systems, remotely controlled early warning systems, etc.; smart food awareness - enabling the consumer with relevant information e.g. concerning safety, availability, health, environmental protection, animal welfare, etc. using chain information systems); enriching the **Eurasian Youth Forum** by a "Black Sea/Caspian Sea Area Initiative" to promote together higher education, innovation and the Bologna process; pushing that initiative of cosmopolitan Youth Organizations also via the **Astana Economic Forum** in Kazakhstan organized among others by the Eurasian Club of Scientists, the Club of Madrid and the G 20. The parties concerned want to use those three activities to create student exchanges by EU-projects like Tempus, Erasmus and Leonardo da Vinci as well as with transatlantic cooperation involving the USAID, USDA and identify the link between higher education, innovation the business community and future employment opportunities and sustainable development in the Eurasia region.

